# Cannabis Essential Oil: A Preliminary Study for the Evaluation of the Brain Effects

**DOI:** 10.1155/2018/1709182

**Published:** 2018-01-17

**Authors:** Nadia Gulluni, Tania Re, Idalba Loiacono, Giovanni Lanzo, Luigi Gori, Claudio Macchi, Francesco Epifani, Nicola Bragazzi, Fabio Firenzuoli

**Affiliations:** ^1^Referring Center for Phytotherapy (CERFIT), Region of Tuscany, Florence, Italy; ^2^Unesco Professorship “Antropologia della Salute-Biosfera e Sistemi di Cura”, University of Genoa, Genoa, Italy; ^3^Center for Integrative Medicine, Careggi University Hospital, Florence, Italy; ^4^Azienda USL 11, Empoli, Italy; ^5^Center IRCCS Don Carlo Gnocchi, Florence, Italy; ^6^Synlab, via della Querciola 12, Sesto Fiorentino, Italy

## Abstract

We examined the effects of essential oil from legal (THC <0.2% w/v) hemp variety on the nervous system in 5 healthy volunteers. GC/EIMS and GC/FID analysis of the EO showed that the main components were myrcene and *β*-caryophyllene. The experiment consisted of measuring autonomic nervous system (ANS) parameters; evaluations of the mood state; and electroencephalography (EEG) recording before treatment, during treatment, and after hemp inhalation periods as compared with control conditions. The results revealed decreased diastolic blood pressure, increased heart rate, and significant increased skin temperature. The subjects described themselves as more energetic, relaxed, and calm. The analysis EEG showed a significant increase in the mean frequency of alpha (8–13 Hz) and significant decreased mean frequency and relative power of beta 2 (18,5–30 Hz) waves. Moreover, an increased power, relative power, and amplitude of theta (4–8 Hz) and alpha brain waves activities and an increment in the delta wave (0,5–4 Hz) power and relative power was recorded in the posterior region of the brain. These results suggest that the brain wave activity and ANS are affected by the inhalation of the EO of* Cannabis sativa* suggesting a neuromodular activity in cases of stress, depression, and anxiety.

## 1. Introduction

The European Pharmacopoeia, sixth Edition (2007), lists 28 essential oils (EOs) [1]. These oils are employed by inhalation and dermal (percutaneous absorption) and oral ingestion in the form of capsules. Industrial hemp (*Cannabis sativa* L. cultivars) is cultivated for fiber and seed production, but has an incredible number of possible applications as ingredient in the cosmetics industry, as flavouring for beverages (food industry), and in medicine. Several studies have been carried out on the cannabinoid content, resin, and seed oil of* Cannabis sativa *L. cultivars, but few studies have focused on the chemical composition and pharmacology of the essential oil extracted from fresh inflorescences and even fewer studies are concerned with its possible uses [2–6].

The hemp essential oil is a complex mixture of many volatile compounds, mainly monoterpenes, sesquiterpenes, and other terpenoid-like substances [7]. The main chemical components are myrcene, *β*-caryophyllene, limonene, *α*-pinene, *β*-pinene, terpinolene, and *α*-humulene. The general properties of these substances include antidepressant, relaxant, anxiolytic, sedative, antimicrobial, and antioxidants [8]. Some researchers studied the antibacterial properties of this oil. These findings show that industrial hemp EOs exhibited good antimicrobial activities, with respect to Gram (+) bacteria such as* Enterococcus hirae, Enterococcus faecium, *and* S. salivarius *subsp.* thermophilus *and against clostridia (in this case only* C. sativa* L. varieties Futura) [9]. The study of Russo describes pharmacological properties of main terpenoids present in industrial hemp EOs [8].

In the research by Bahia et al., they reported that *β*-caryophyllene may be useful in treating anxiety and depression. Moreover they demonstrated the effect of *β*-caryophyllene and its underlying mechanism in a CB2 receptor-dependent manner in mice [10]. These *β*-caryophyllene's properties may explain why Cannabis users often cite relief of anxiety and depression as reason for their use. But, presently, the effects of hemp EOs inhalation on the brain in humans have not been studied and there are no studies on the possible therapeutic use. However, these studies support that hemp EOs inhalation can have a relaxing effect on the nervous system. Thus, this study is the first to focus on aspects such as brain wave activity and ANS parameters such as heart rate, blood pressure, respiratory rate, and skin temperature, as well as the assessment of mood states through comparative measures. Moreover gas chromatography characterization of hemp EOs was conducted.

## 2. Materials and Methods

### 2.1. Essential Oil Analysis

Hexane used for preparing working solution was purchased from Carlo Erba (Rodano, MI, Italy), while the linear n-hydrocarbons (C9–C40) were purchased from Sigma-Aldrich.

The EO used in this study is an extract of* Cannabis sativa L*. (Cannabaceae;* hemp*) purchased from Assocanapa Association (Carmagnola, TO, Italy). The EO was obtained from fresh leaves and inflorescences of* Cannabis sativa L*. were collected and steam distilled by Assocanapa Association, as given by the Italian Official Pharmacopoeia (2,5 L water distilled, 2 h in Clevenger-type apparatus). The Cannabis EO (CEO) yield was 0,11% v/w. CEO and EOs dilutions were stored at 4°C before use. Gas chromatography/electronic ionization mass spectrometry (GC/EIMS) and gas chromatography/Flame Ionization Detector (GC/FID) analyses were accomplished using an HP‐5890 Series II instrument equipped with HP-WAX and HP-5 capillary columns (30 *μ*m × 0.25 mm, 0.25 *μ*m film thickness), detector dual FID, working with the following temperature programme: 60°C for 10 min, ramp of 5°C/min to 220°C; injector and detector temperatures 250°C; carrier gas nitrogen (2 ml/min); detector dual FID; split ratio 1 : 30; injection of 0.5 *μ*l. For both columns, components were identified by comparing their retention times with those of pure authentic samples and by means of their linear retention indices (l.r.i.) [11, 12] relative to the series of n-hydrocarbons. The relative proportions of the EO constituents were percentages obtained by FID peak-area normalization, all relative response factors being taken as one. GC/EIMS analyses were performed with Varian CP‐3800 gas chromatograph (Variant, Inc. Palo Alto CA) equipped with a DB-5 capillary column (Agilent Technologies Hewlett-Packard, Waldbronn, Germany, 30 m × 0.25 mm; coating thickness 0.25 *μ*m) and a Varian Saturn 2000 ion trap mass detector. Analytical conditions were as follows: injector and transfer line temperatures 220 and 240°C, respectively; oven temperature programmed from 60 to 240°C at 3°C/min; carrier gas helium at 1 ml/min; injection of 0.2 *μ*l (10% hexane solution); split ratio 1 : 30. Identification of constituents was based on comparison of the retention times with those of authentic samples, comparing their l.r.i. relative to the series of n-hydrocarbons (C9–C40), and on computer matching against commercial (NIST 98 and ADAMS 95) and home-made library mass spectra built up from pure substances and components of known oils and MS literature data [13, 14].

The percentage compositions were computed from the GC peak areas. Moreover, the molecular weights of the all identified substances were confirmed by GC/CIMS, using MeOH as CI ionizing gas. Analysis of the essential oils identified 35 constituents (Table 1), accounting for 97.6% of the total oils (only compounds >0,1% are reported in Table 1). Monoterpene hydrocarbons represented the 57.2% of total volatiles and myrcene was the main constituent (22.9%). Sesquiterpenes hydrocarbons represented the second class of volatiles (34.3%) with the most abundant being *β*-caryophyllene (18.7%).

### 2.2. Subjects

Five healthy volunteers (3 males and 2 females) aged 30 to 57 years (mean age 40,8 ± 12,19 years) and with body mass index (BMI) between 19,05 and 34,60 kg/m^2^ (mean BMI 26,986 ± 7,18 kg/m^2^) participated in this study. Demographic data of the participants is presented in Table 2. Only five volunteers were available for the experimental session preprogrammed; other recording experimental sessions were not made because environmental parameters would not be reproducible and comparable. None of the subjects had cardiovascular disease, they did not exhibit any symptoms of upper respiratory infection, and women were not to be in their menstrual period on the day of the tests. Two subjects were smokers and one of the male subjects had a slight headache at the time of the experiment. All experimental procedures were followed with the strict ethical standards formulated in the Helsinki Declaration of 1964 that was revised in 2000 and all the subjects have participated in the study after signing the informed consent. The study was conducted in private healthcare facilities outside the network of the Regional Health System. Therefore, any ethical and managerial assumption is rooted in the direct relationship between the patient, who has released the relative consent, and the host structure.

### 2.3. Methods

**Figure 1:**
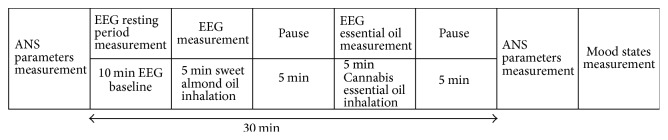
Experimental protocol of EEG, ANS, and mood states measurements divided into 8 blocks: ANS parameters recording; EEG recording in baseline condition (10 min), EEG in placebo condition (1 ml sweet almond oil inhalation, 5 min); pause (5 min); EEG* Cannabis* EO inhalation (1 ml, 5 min); pause (5 min); ANS parameters recording; and finally mood states measurement.

One milliliter of sweet almond oil (SAO, base oil, purchased from Carlo Erba) was used for control condition as placebo and one milliliter of CEO was administered according to the protocol described in Figure 1. The sweet almond oil was administered with gauze and participants were asked to inhale simultaneously with both nostrils. The same procedure was also adopted for the CEO. In accordance with previous studies, it has been found that the pleasantness of the aroma of the oil could alter autonomic activity [15, 16]. As a result of these facts, the subjects were asked to inhale SAO and CEO to assess the pleasantness or less pleasantness of the aroma.

### 2.4. ANS and Mood State Measurement

The mood state and ANS parameters of the subjects such as blood pressure, heart rate, skin temperature, and respiratory rate were recorded simultaneously. The ANS parameters were measured manually. While the evaluation of the changes of moods was made through a subjective evaluation of the participants using a list of 8 terms selected for their relevance to describe affective feelings induced by odors and mood state after inhalation of CEO: anxiety, calm, hungry, hilarity, fatigue, apathy, energy, and heavy eye [17–19].

### 2.5. EEG Recording

A set of 21 electrodes with an additional ground electrode and a reference electrode were placed onto the subject's head with electrocap in accordance with the international 10–20 system at F1, F2, F7, F3, Fz, F4, F8, T3, C3, Cz, C4, T4, T5, P3, Pz, P4, T6, O1, and O2. Mizar 40 EBNeuro-Firenze was used as the recording system. Sampling rate was set at 512 Hz; the HF filter was set at 70 Hz; time constant 0,3; range −/+4,1 mV. The relative power spectrum of the respective frequency bands was expressed as follows: delta (0,5–4 Hz), theta (4–8 Hz), alpha (8–14 Hz), and beta (14–30 Hz). Furthermore, the beta wave was further categorized as beta 1 or low frequency beta (14–18 Hz) and beta 2 or high frequency beta (18,5–30 Hz) activities.

### 2.6. Experimental Protocol/Procedure

All the steps in this experiment were similarly conducted as the previous study recorded on the effects of rosemary oil inhalation [20]. All activities were conducted in a quiet room and the subjects were seated in a comfortable chair. The ANS electrodes were attached to the appropriate positions. The ANS parameters: heart rate, skin temperature, respiratory rate, and systolic and diastolic blood pressure were recorded at the beginning of the experiment before resting (baseline) EEG measurement and after CEO inhalation.

The experiment consisted of three trials: first session served as a baseline (resting period) and took ten minutes. The second and the third sessions took five minutes each.

The first session baseline EEG recording was done with both eyes opened and eyes closed, respectively. In the second session, SAO was inhaled by the subjects. In the third session, CEO was applied and mood state was measured after inhalation. EEG was recorded for five minutes during SAO inhalation and after five minutes of rest it was recorded again for five minutes during CEO inhalation. After the recordings, the subjects were asked to give their preference and impression of the odors presented and of their moods (Figure 1).

### 2.7. Statistical Analyses

The MedCalc statistics for biomedical research software version 16.2.1 was done for data analysis of the effects of CEO on physiological reactions and mood states, before and after the hemp inhalation. A nonparametric Kruskal Wallis signed rank test was used for EEG data analysis and Friedman test was performed to determine whether the activity changed significantly in any of frequency bands in P4-O2 and P3-O1 brain region. A paired *t*-test was carried out on data concerning the subjects' blood pressures, heart rates, skin temperatures, and respiratory rates. A *p* value < 0,05 was considered significant. A percentage evaluation was done for the mood states.

## 3. Results and Discussion

In the present research, CEO was administered by inhalation to healthy subjects and we examined the effects of the oil on the human nervous system. Brain wave activity and ANS parameters (blood pressure, heart rate, respiratory rate, and skin temperature) were recorded as the indicators of the arousal level of the nervous system. In addition, we studied the effects of CEO on moods by performing subjective self-evaluation in order to assess the arousal levels.

### 3.1. Autonomics Nervous System Parameters

Inhalation of CEO has been correlated with changes in ANS parameters and skin temperature significantly increased (*p* < 0,05). The data of various ANS parameters were compared during rest (control) and CEO inhalation as shown in Table 3. In 60% of subjects heart rate had increased during CEO exposure. In contrast, diastolic blood pressure had decreased in 80% of subjects. But these data did not reach statistical change. These changes of the ANS parameters indicated an involvement of the autonomic nervous system and parasympathetic nervous system (PNS). The stimulatory effects on the ANS and PNS may be explained through the presence of monoterpenes (limonene and *α*-pinene) that are present in CEO.

The *α*-pinene inhibits acetylcholinesterase [21], which results in the activation of the PNS, and this might be responsible for the reduction in diastolic blood pressure. While the stimulatory effects on the sympathetic system determined by limonene might be responsible for the increase in heart rate and skin temperature [22].

### 3.2. Emotional Parameters

All subjects found the CEO pleasant. The alterations of mood states after exposure to CEO are shown in Table 4. The subjects felt more calm, relaxed, and energetic, were in a good mood, and had increased feeling of hunger and the subject with headache had no more pain. These results indicate that CEO inhalation increases the level of relaxation and general well-being as assessed through our test subjects' self-evaluation. This relaxing and anxiolytic effect on ANS could be explained by the abundance of limonene, myrcene, and *β*-caryophyllene, main components of the EO. Several studies in animals and humans suggest that the limonene can be a powerful anxiolytic agent via 5-HT. Also, limonene demonstrated antistress effects on the brain of rats. Bahia and colleagues found that b-caryophyllene has an anxiolytic and antidepressant activity in a CB2 receptor-dependent manner [10, 22].

Myrcene, the main component of CEO, has a sedative, analgesic, and relaxing activity [23, 24]. Thus, these results confirm that CEO contains mood-elevating bioactive components that prove to be beneficial to its users.

### 3.3. EEG Parameters

EEG spectral analysis was done with quantitative evaluation of windows on 2 seconds with Interpolation Algorithm Rectangular.

Five frequency bands were evaluated (delta, theta, alpha, beta 1, and beta 2) and values of power (*μ*V^2^), amplitude (*μ*V), relative power (*μ*V^2^), and mean frequency were calculated during rest, during SAO inhalation, and during CEO inhalation states. The studied areas were divided into the right posterior area (P4-O2) and left posterior area (P3-O1) brain regions. The data recording shows an alteration of EEG during exposure to the CEO. There were noticeable changes of band power, amplitude, and relative power in alpha, theta, delta, and beta waves as reported in Tables 5, 6, 7, and 8 (mean and median value).

**Figure 2:**
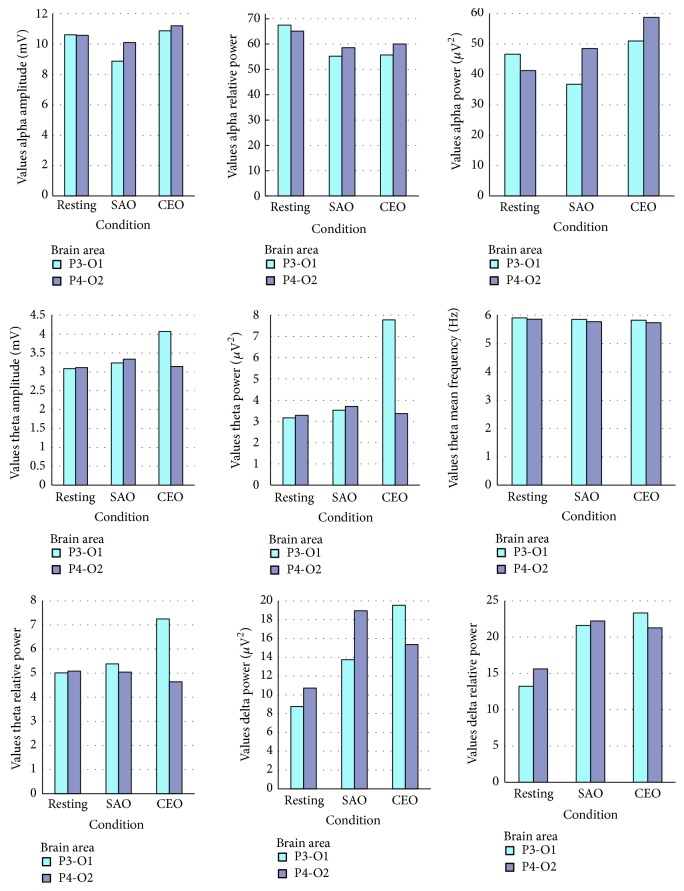
Each panel shows the mean of power, relative power, mean frequency, and amplitude values of alpha and theta activities for the resting and sweet almond oil (SAO) and cannabis essential oil (CEO) inhalation states. The theta and delta waves increased mostly in P3-O1.

**Figure 3:**
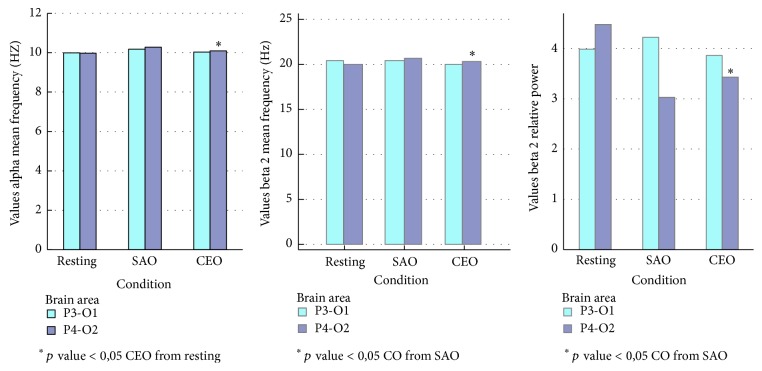
Values of mean frequency and power relative to alpha and beta 2 with significant difference in P4-O2 area of brain.

During the CEO inhalation the power, the relative power, and amplitude of the alpha waves in both brain regions were increased and mean frequency for alpha significantly increased (compared with SAO) in P4-O2 brain area (*p* < 0,05). The power and relative power changes in the theta and delta waves in the left posterior brain region were also increased. In contrast, the power in the delta wave in the right posterior brain region was decreased. A significant decrease was observed in the case of the beta 2 wave's relative power (compared with resting condition) and mean frequency (compared with SAO) in P4-O2 (*p* < 0,05), Figures 2 and 3. The present research shows the effects of CEO inhalation on brain waves. This research showed that alpha (8–13 Hz) and theta (4–8 Hz) activity increased during CEO exposure in the posterior regions, and mostly left posterior area P3-O1 brain regions. Moreover, alpha mean frequency increased significantly in P4-O2 region. These results show concordance with the past EEG studies on the effects of odors which demonstrated increased alpha activity by administration of several EOs such as lavender, chamomile, *α*-pinene, and limonene oil [25–28]. Instead alpha activity is attenuated under emotional tension and stress condition [29].

The EEG evidence of relaxation can be seen in various practices such as meditation, yoga, Qigong, and mindfulness [30, 31]. The study among people meditating can demonstrate similar EEG changes with CEO inhalation, which presented as increase in theta and alpha activities in the brain during meditation [32, 33]. In addition, the studies of Aftanas [34–37] show that during meditation there is also release of hormones such as melatonin, serotonin, and cortisol. These results lend support that increases in theta and alpha waves activity cause a range of general relaxation and anxiolytic effects on the brain and also some possible decreases of pain. Thus, the data recorded after CEO inhalation shows relaxation and anxiolytic effects on the brain at level of the ANS, CNS, and mood states. At level of mood states a feeling of calm, relaxation, and decreased anxiety was recorded indicating the involvement of the limbic system.

The changes in ANS parameters (heart frequency, skin temperature, and diastolic blood pressure) can be explained by the *α*-pinene activity on the parasympathetic system and limonene activity on the sympathetic system action. Komiya et al. [22] found that limonene increases serotonin in the prefrontal cortex, and dopamine (DA) in hippocampus mediated via 5-HT1A. This determines the direct activation of the sympathetic system. At levels of CNS activity, alpha and theta waves increased indicating a relaxing effect and antidepressant and antianxiety effect due to the *β*-caryophyllene and limonene. The analgesic action of CEO on the subject with headache may be explained by increase of alpha and theta waves and abundance of terpenes such as myrcene, limonene, and *β*- caryophyllene.

## 4. Conclusions

The small study population is a limitation of this study but it is however a preliminary study. Further studies of the effect of CEO on the brain are needed with a wider sample in order to have a greater number of significant data. However, the results lend some support for including CEO in a perspective integrated therapy aimed at relieving stress or depression.

The results suggest the occurrence of the positive relaxation and anxiolytic effects of CEO. These findings provide evidence that brain wave activity autonomic nervous system response and mood states were affected by CEO.

## Figures and Tables

**Table 1:** GC-MS results of the essential oil extracted from hemp inflorescences (*Cannabis sativa *L. var. monoica).

Constituents	l.r.i.^∧^	Percentage
*α*-Pinene	941	7,7
Camphene	955	0,2
Sabinene	978	0,2
*β*-Pinene	982	3,7
Myrcene	993	22,9
*α*-Phellandrene	1007	0,3
*δ*-3-Carene	1010	0,6
*α*-Terpinene	1020	0,3
*p*-Cymene	1028	0,5
Limonene	1033	3,9
1,8-Cineole	1035	0,2
(*Z*)-*β*-Ocimene	1042	0,7
(*E*)-*β*-Ocimene	1053	3,9
*γ*-Terpinene	1063	0,3
Terpinolene	1090	12,0
Linalool	1101	0,3
*p*-Cymen-8-ol	1184	0,5
*α*-Terpineol	1192	0,2
Carvacrol	1301	0,2
(*Z*)-Caryophyllene	1406	0,7
*β*-Caryophyllene	1419	18,7
*trans*-*α*-Bergamotene	1438	1,5
*α*-Humulene	1455	6,2
9-*epi*-Caryophyllene	1468	2,3
*γ*-Muurolene	1478	0,2
*β*-Selinene	1487	1,6
*α*-Selinene	1495	1,5
*β*-Bisabolene	1508	0,4
*trans*-*γ*-Cadinene	1514	0,2
*δ*-Cadinene	1524	0,2
Selina-3,7(11)-diene	1544	0,6
Germacrene B	1557	0,2
Caryophyllene oxide	1582	3,7
Humulene oxide II	1607	1,0

Monoterpene hydrocarbons		57,2
Oxygenated monoterpenes		1,4
Sesquiterpene hydrocarbons		34,3
Oxygenated sesquiterpenes		4,7

*Total identified*		*97,6*

Percentages are obtained by FID peak-area normalization, all relative response factors being take as one (HP-5) column. Mean of three analyses. ^∧^Linear retention indices (HP-5 column) relative to the series of n-hydrocarbons.

**Table 2:** Demographic data for subjects.

Parameters	Subjects (^*∗*^M, ^*∗*^F)					Minimum	Maximum	Mean	SD
	M1	M2	M3	F1	F2				
Age	57	30	50	30	37	30	57	40,8	12,19
Weight (Kg)	90	100	95	55	50	50	100	78	24,04
Height (cm)	176	170	172	165	162	162	176	169	5,56
BMI (Kg/m^2^)	29,05	34,6	32,11	20,2	19,05	19,05	34,6	26,996	7,18

^*∗*^M: Male; ^*∗*^F: Female.

**Table 3:** In detail the values, mean, and standard deviation of ANS parameters for resting condition and after Cannabis oil inhalation.

Subjects (^*∗*^M, ^*∗*^F)	Demographic data				ANS parameters									
	Age	Weight (kg)	Height (cm)	BMI (kg/m)	Resting					Cannabis essential oil				
					Systolic blood pressure (mmHg)	Diastolic blood pressure (mmHg)	Heart rate (bmp)	Respiratory rate (bpm)	Skin temperature (°C)	Systolic blood pressure (mmHg)	Diastolic blood pressure (mmHg)	Heart rate (bmp)	Respiratory rate (bmp)	Skin temperature (°C)
M1	57	90	176	29,5	140	90	88	14	36,5	150	95	88	13	36,5
M2	30	100	170	34,6	120	80	66	18	36,3	115	75	74	14	36,7
F1	30	55	165	20,2	88	65	72	14	36,6	85	60	72	15	36,8
M3	50	95	172	32,11	125	95	62	15	36,2	120	80	75	15	36,4
F2	37	50	162	19,0	110	70	88	18	36	108	65	94	20	36,4
MEAN	40,8	78	169	26,9	116,6	80	75,2	15,8	36,3	115,6	75	80,6	15,4	*35,6*
SD (±)	12,2	24,0	5,6	7,2	19,3	12,7	12,2	2,1	0,24	23,4	13,7	9,8	2,7	*0,18* ^*∗*^

^*∗*^ *p* < 0,05 significance when compared to resting condition; ^*∗*^M: Male; ^*∗*^F: Female.

**Table 4:** Percentages of emotional states scores after cannabis essential oil inhalation.

Subjects (^*∗*^M, ^*∗*^F)	Demographic date				Emotional states (self-evaluation)							
	Age	Weight (kg)	Height (cm)	BMI (kg/m)	Anxiety	Calm	Hunger	Hilarity	Heaviness eye	Tiredness	Apathy	Energy
M1	57	90	176	29,5	Decreased	-	Increased	Increased	-	-	Increased	*Increased*
M2	30	100	170	34,6	Decreased	Increased	Increased	-	Increased	-	-	Decreased
F1	30	55	165	20,2	-	Increased	Increased	-	Increased	-	Increased	Decreased
M3	50	95	172	32,1	-	Increased	Increased	-	Increased	-	-	Decreased
F2	37	50	162	19,0	-	Increased	-	Increased	-	-	-	*Increased*

%					40%	80%	80%	40%	60%	-	40%	60%/*40%*

^*∗*^M: Male; ^*∗*^F: Female.

**Table 5:** Mean and median power values and *p* value for resting condition, sweet almond oil inhalation, and cannabis essential oil inhalation.

Brain area	Resting	Sweet almond oil	Cannabis essential oil	*p* value
Theta power (*µ*V^2^)				
P4-O2	3,286 (2,77)	3,704 (2,75)	3,366 (3,36)	0,268
P3-O1	3,166 (3,19)	3,522 (3,16)	7,776 (5,21)	0,497
Alpha power (*µ*V^2^)				
P4-O2	41,266 (38,95)	48,592 (39,41)	58,788 (40,17)	0,599
P3-O1	46,698 (48,45)	36,8 (30,88)	51,036 (51,26)	0,268
Delta power (*µ*V^2^)				
P4-O2	10,734 (4,68)	18,96 (12,23)	15,36 (15,72)	0,167
P3-O1	8,768 (8,4)	13,756 (8,14)	19,542 (17,09)	0,268
Beta 1 power (*µ*V^2^)				
P4-O2	5,816 (4,61)	8,778 (5,53)	9,05 (7,12)	0,849
P3-O1	6,402 (8,19)	8,01 (7,61)	8,268 (8,4)	0,849
Beta 2 power (*µ*V^2^)				
P4-O2	3,086 (4,05)	2,47 (3,08)	2,83 (3,1)	0,268
P3-O1	2,714 (2,64)	2,918 (2,3)	3,734 (4,14)	0,849

SAO: sweet almond oil; CEO: cannabis essential oil.

**Table 6:** Mean and median relative power values and *p* value for resting condition, sweet almond oil inhalation, and cannabis essential oil inhalation.

Brain area	Resting	Sweet almond oil	Cannabis essential oil	*p* value
Theta PotR				
P4-O2	5,082 (4,06)	5,048 (4,61)	4,642 (3,78)	0,268
P3-O1	5,01 (5,53)	5,386 (5,67)	7,248 (6,32)	0,497
Alpha PotR				
P4-O2	65,098 (69,39)	58,608 (60,74)	60,042 (63,23)	0,497
P3-O1	67,488 (68,22)	55,212 (56,46)	55,746 (50)	0,268
Delta PotR				
P4-O2	15,64 (14,32)	22,254 (24,25)	21,284 (25,16)	0,497
P3-O1	13,234 (13,36)	21,606 (20,48)	23,34 (25,39)	0,497
Beta 1 PotR				
P4-O2	8,224 (8,4)	9,444 (7,98)	9,09 (9,89)	1,00
P3-O1	9,106 (7,57)	12,054 (8,59)	8,318 (9,4)	0,849
Beta 2 PotR				
P4-O2	4,482 (2,97)	3,028 (2,82)	3,432 (2,48)	*0,05308* ^**∗**^
P3-O1	3,986 (2,84)	4,226 (3,89)	3,862 (3,54)	0,849

SAO: sweet almond oil; CEO: cannabis essential oil. ^*∗*^CEO differs from resting with *p* value < 0,05.

**Table 7:** Mean and median amplitude power values and *p* value for resting condition, sweet almond oil inhalation, and cannabis essential oil inhalation.

Brain area	Resting	Sweet almond oil	Cannabis essential oil	*p* value
Theta Amp				
P4-O2	3,11 (2,89)	3,336 (3,06)	3,142 (3,12)	0,268
P3-O1	3,082 (3,13)	3,234 (3,18)	4,066 (3,99)	0,497
Alpha Amp				
P4-O2	10,574 (10,06)	10,104 (9,63)	11,204 (10,07)	0,599
P3-O1	10,614 (10,37)	8,88 (8,4)	10,872 (11,65)	0,073
Delta Amp				
P4-O2	4,312 (3,63)	5,564 (5,25)	5,45 (5,55)	0,073
P3-O1	4,148 (4,4)	5,246 (4,43)	6,06 (5,53)	0,167
Beta 1 Amp				
P4-O2	4,946 (4,76)	5,32 (5,2)	5,548 (5,87)	0,599
P3-O1	5,06 (5,85)	5,37 (6,07)	5,592 (6,36)	0,849
Beta 2 Amp				
P4-O2	3,526 (4,14)	3,296 (3,76)	3,458 (3,72)	0,268
P3-O1	3,408 (3,56)	3,432 (3,56)	3,838 (4,28)	0,958

SAO: sweet almond oil; CEO: cannabis essential oil.

**Table 8:** Mean and median mean frequency values and *p* value for resting condition, sweet almond oil inhalation, and cannabis essential oil inhalation.

Brain area	Resting	Sweet almond oil	Cannabis essential oil	*p* value
Theta Mean*F*				
P4-O2	5,854 (5,83)	5,772 (5,78)	5,732 (5,84)	0,497
P3-O1	5,906 (5,930)	5,848 (5,89)	5,82 (5,94)	0,849
Alpha Mean*F*				
P4-O2	9,982 (9,99)	10,274 (10,28)	10,094 (10,04)	*0,00066* ^*∗*^
P3-O1	9,984 (9,96)	10,178 (10,13)	10,026 (9,84)	0,268
Delta Mean*F*				
P4-O2	1,572 (1,61)	1,464 (1,41)	1,474 (1,3)	0,849
P3-O1	1,452 (1,46)	1,428 (1,48)	1,508 (1,38)	0,958
Beta 1 Mean*F*				
P4-O2	14,182 (14,3)	13,89 (14,16)	14,092 (14,58)	0,167
P3-O1	14,026 (14,19)	14,082 (14,34)	14,256 (14,52)	0,599
Beta 2 Mean*F*				
P4-O2	20,026 (20,07)	20,592 (20,57)	20,266 (20,31)	*0,00332* ^*∗*^
P3-O1	20,34 (20,55)	20,348 (20,54)	19,982 (19,91)	0,0731

SAO: sweet almond oil; CEO: cannabis essential oil. ^*∗*^CEO differs from SAO with *p* value < 0,05.

## Abbreviations

ANS: Autonomic nervous system
CNS: Central nervous system
CB2: Cannabinoid receptor type 2
EEG: Electroencephalography
EO: Essential oil
CEO: Cannabis essential oil
SAO: Sweet almond oil
GC/FID: Flame Ionization Detector
GC/EIMS: Gas chromatography electronic ionization mass spectrometry
PNS: Parasympathetic nervous system
THC: Tetrahydrocannabinol.
